# Community cervical cancer screening and precancer risk in women living with HIV in Jos Nigeria

**DOI:** 10.1186/s12889-024-17739-z

**Published:** 2024-01-16

**Authors:** F. A. Magaji, M. I. Mashor, S. A. Anzaku, A. R. Hinjari, N. T. Cosmas, B. V. Kwaghe, J. M. Ali, Elizabeth N. Christian, A. S. Sagay, Ariel Chandler, Imran Khan, Robert L. Murphy, Lifang Hou, J. Musa

**Affiliations:** 1 Gynecologic-Oncology Division, Department of Obstetrics and Gynecology, University of Jos, Jos, Nigeria; 2 College of Health Sciences, University of Jos, Jos, Nigeria; 3 Department of Histopathology, Jos, Nigeria; 4 Bingham University Teaching Hospital, Jos, Nigeria; 5 Department of Medical Microbiology, Jos, Nigeria; 6 Department of Anatomic Pathology and Forensic Medicine, Jos University Teaching Hospital, Jos, Nigeria; 7 Robert J. Havey, MD Institute for Global Health, Chicago, IL USA; 8 Northwestern University Feinberg School of Medicine, Chicago, IL USA; 9 Program Department Health Analytics, School of Professional Studies, Northwestern University, Chicago, IL USA; 10 Center for Global Oncology, Institute for Global Health, Chicago, IL USA; 11 Department of Preventive Medicine, Division of Cancer Epidemiology & Prevention, Chicago, IL USA

**Keywords:** Adult HIV-clinic, Indigent women, Pap test, Cervical precancer and cancer, Jos-Nigeria

## Abstract

**Background:**

High HIV prevalence, and lack of organized screening for the indigent population receiving care and treatment within HIV clinics in low-resource settings increases cervical cancer incidence. We sought to determine predictors of cervical precancer in women living with HIV and receiving cervical cancer screening in Jos, Nigeria.

**Methods:**

A cross-sectional study of women living with HIV and receiving care and treatment in adult HIV/AIDS clinics in Jos-Metropolis, Nigeria between June 2020 and April 2023. Ethical approvals were obtained from the ethics committee in Jos, Nigeria and Northwestern University IRB, USA. Informed consent was obtained from eligible participants, and data on socio-demographics, cancer risk factors, and cytology reports were collected. The outcome variables were cervical precancer lesions. The independent variables were prior Pap smear status, socio-demographics, income, educational, and other reproductive health factors. Descriptive statistics was done to obtain means ± sd, frequencies, and percentages for the variables. Univariate and bivariate analyses were done to determine predictors of cervical dysplasia. Analyses were performed using R software.

**Results:**

Of 957 women screened, 570 were living with HIV and 566 women had cytology report and were included in the final analysis. The mean age was 45.08 ± 8.89 years and 81.6% had no prior evidence of Pap test (under-screened). Prevalence of cervical dysplasia was 24% (mild and severe dysplasia were 12.9% and 11.1%, respectively). Age above 45 years (aOR = 3.48, *p* = 0.009), postmenopausal status (aOR = 7.69, *p* = 0.000), and women with no history of prior IUCD use (aOR = 5.94, *p* = 0.0001), were predictors for severe dysplasia. Women who had history of STI (aOR = 0.17, *p* = 0.000), prior use of IUCD (aOR = 0.32, *p* = 0.004), prior use of condom (aOR = 2.50, *p* = 0.003) and had co-morbidities (aOR = 0.46, *p* = 0.009) were more likely to have had a Pap test in the past.

**Conclusions:**

The majority of indigent women receiving care at HIV clinics had their first Pap test screening, and lack of organized screening among older and post-menopausal women with HIV, puts women at a higher risk of developing severe cervical precancer lesions.

## Background

Globally, cervical cancer is the fourth most common malignancy in women with an estimated 604,127 new cases, and 341,831 deaths recorded in 2020 alone. Over 85% of the new cases and mortalities occurred in low-resource countries [1, 2]. Women living with HIV (WLWH), the majority of whom reside in sub-Saharan Africa (SSA), are at increased risk of cervical cancer due to higher prevalence and persistence of human papillomavirus (HPV), the causative agent [3]. Compared to HIV negative women, WLWH have a shorter duration from HPV infection to development of precancerous lesions, and have a 2 to 12 fold increased risk of developing cervical cancer, making prevention efforts an urgent priority among WLWH [4].

The global distribution of country-specific HIV prevalence matches the global distribution of cervical cancer incidence and mortality [5, 6]. More than 50% of the people living with HIV (PLHIV) in SSA countries are women and girls [3]. Nigeria, second only to South Africa with the highest number of PLHIV, is estimated to have over 53 million women at risk of cervical cancer, and cervical cancer screening (CCS) covers less than 9% of the general population [7]. The high prevalence of HIV, limited availability of HPV vaccination, lack of organized screening programs and treatment of precancerous cervical lesions are the contributing factors for the high cervical cancer incidence and mortality in low-resource settings [8, 9].

In 1993, following the classification of cervical cancer as an AIDS-defining malignancy, the Infectious Disease Society of America (IDSA) recommended CCS with Pap test (Papanicolaou) twice in the first year of HIV diagnosis and annually thereafter. This is a more frequent screening interval than the triennial screening recommended for HIV negative women [4]. The majority of the HIV control interventions in low-resource settings are vertical programmes, with support focusing only on HIV care, antiretroviral therapy (ART) access and viral load suppression, and non to address cervical cancer among WLWH [10]. The limited access to CCS services within HIV clinics exposes WLWH to preventable cervical cancer-related incidence and mortality thereby eroding the hard-fought gains from access to HIV treatment [11, 12]. Therefore, the study sought to determine predictors of cervical dysplasia in WLWH receiving care and treatment in adult HIV care clinics in Jos.

## Methods

### Study site

This study was conducted in Jos-metropolis at the HIV/AIDS comprehensive treatment clinic of Jos University Teaching Hospital (JUTH), and Bingham University Teaching Hospital (BHUTH), with referral from Plateau State Specialist Hospital (PSSH), and Our Lady of Apostles Hospital (OLA), all in Jos Metropolis, Nigeria.

### Study population

The study population were women living with HIV and receiving ART in the HIV care and treatment clinics at the study sites within Jos Metropolis.

### Study recruitment and enrollment

The project offered opportunistic cervical cancer screening services to eligible female population, who were at least 21 years for those living with HIV infection, sexually active, not pregnant, no history of hysterectomy, and who have not had cervical screening test in the last one year.

### Recruitment

We sequentially recruited and enrolled eligible women for all the study objectives from the study population. Written informed consent for the study was obtained from the study participants prior to enrollment into the study. All information obtained were coded and kept confidential. Study participants who declined to participate in the study were provided cervical screening services and their data was not included in this analysis.

### Study design

This was a cross-sectional study conducted between June 20, 2020 and April 30, 2023. Institutional Review Board (IRB) for the study was obtained from the Health Research Ethical Review Committees of JUTH and BHUTH locally in Nigeria and Northwestern University, Chicago, Illinois, USA.

### Study procedure: collection of data and sample

At entry, data on socio-demographic, cigarette smoking, parity, and use of hormonal contraception, awareness of CCS, previous CCS, were collected through the questionnaire. Height and weight were measured to calculate body mass index (BMI) of the study participants. Study examination took place in a private room equipped with a gynaecologic examination bed in the clinic. Participants was positioned in the dorsal lithotomy position. After the introduction of a sterile speculum, a cytology brush was inserted in to the transformation zone of the cervix and rotated to take cervical epithelial cells. A smear was made on a glass slide and fixed for preparations by the Technologist. In this study, three cytopathologists read the pap slides. To ensure quality control in reading the pap smear slides, every slide was read by at least two cytopathologists and where there was any ambiguity or difference in report between the two cytopathologists, a third cytopathologist read the same pap smear slide (tie breaker). All the cytopathologists followed the Standardized framework for reading the prepared pap smear and reported based on the 2001 Bethesda System. Clinical data and the cytology reports of the Pap tests were collected using a questionnaire adopted from the existing data tool for the operation stop cervical cancer project [7], the records from the questionnaire were entered into the institutional electronic REDCap database hosted at the University of Jos [13–15].

### Operational definitions

The cervical cytology screening outcomes were reported according to the Bethesda 2001 reporting system [16]. The system classified cervical cytology result by initially triaging into specimen adequacy as satisfactory or unsatisfactory for evaluation. Specimen satisfactory for evaluation are further classified into negative for intraepithelial lesion or malignancy (NILM) for which no epithelial abnormality was identified. The epithelial cell abnormalities (cervical precancer lesions) were classified as Atypical squamous cells, which are qualified as “of undetermined significance (ASC-US)” or “cannot exclude HSIL” (ASC-H). The squamous intraepithelial lesion (SIL) was subdivided into low-grade SIL (LSIL) and high-grade SIL (HSIL) for reporting the non-invasive squamous cervical abnormalities. The NILM, atypical squamous cell and SIL groups were further classified into Normal for NILM, Mild dysplasia for ASC-US and LSIL, and severe dysplasia for ASC-H and HSIL.

The key dependent variables were cervical cytology reports following Pap test and history of prior Pap test (screened) for cervical cancer of women living with HIV. Under-screened status (first Pap smear in the index study) is when there is no evidence of prior Pap test since diagnosis with HIV infection, Screened status is when there is at least one previous Pap test in the past since diagnosis with HIV infection.

The independent variables included socio-demographic characteristics like age, income status, completed level of education, smoking history, alcohol consumption, reported life time number of sexual partners, age at first pregnancy and live births, number of live births, use of oral contraceptive pills and Intrauterine Contraceptive Devices (IUCD), awareness of screening and previous CCS.

### Statistical analysis

#### Descriptive statistics

Continuous variables were described as means and standard deviations (sd) while categorical variables were described as frequencies with percentages and compared using Chi square test and Fisher exact when appropriate. We also compared baseline characteristics of the sample with the primary outcome.

#### Analysis for cervical dysplasia and under-screened women as primary outcomes

We estimated the relative proportions of the various categories of cytology outcomes at CCS reported according to the Bethesda system and corresponding *p*-value. For analytic convenience and ease of interpretation, we categorized the cytology report into three groups in the first instance as follows: NILM as category 1 (reference category); ASC-US and LSIL (mild cervical dysplasia) as category 2; and ASC-H, Atypical Glandular Cells (AGC), and HSIL (severe cervical dysplasia) as group 3. In the second instance, we classified: NILM (normal) as group “a” and combine mild and severe cervical dysplasia (cervical dysplasia) as group “b”. We estimated the proportions for each of these sub-classifications. We compared the baseline socio-demographic characteristics of the study sample by cervical cytology groups using the Pearson’s chi square or Fisher’s exact test where applicable and obtain corresponding *p*-values. The same process was repeated for the history of Pap test (screened) women as primary outcome.

#### Bivariate logistic regression

We performed bivariate logistic regression to obtain the odds ratios of the association between baseline socio-demographic variables and abnormal cervical cytology, dummy variables were created for the sub-classification NILM as reference. We performed separate bivariate logistic regression to estimate the odds of having mild cervical dysplasia (category 2) and severe cervical dysplasia (category 3), respectively at socio-demographic characteristics and other independent variables in the study sample. For each of these categories, we estimated the unadjusted odds ratio (OR), 95% confidence intervals (CI), and the corresponding *p*-values. This was also repeated for the history of Pap test (screened) women as primary outcome.

#### Multivariate logistic regression

We built a multivariate logistic regression model to assess the independent effect of socio-demographic characteristics, income status, age, educational level, awareness of CCS, previous CCS on the likelihood of an abnormal cervical cytology outcome report at CCS. As in the bivariate logistic regression model, we used the second sub-classification having group “a” (normal) as reference. We performed multivariate logistic regression for group “b” (cervical dysplasia). In selecting the best predictive model, we used the foreword selection method with Akaike Information Criterion (AIC), Bayesian Information Criterion (BIC) and the overall changes in the model effect to select the covariates that remain in each of the final predictive models. We estimated the 95% confidence intervals for each of these measures of association, and the corresponding *p*-values. The same process was repeated for the history of Pap test (screened) women as primary outcome.

## Results

Out of the 957 women screened for cervical cancer during the community outreach project, 570 women were living with HIV infection, 4 women had unsatisfactory Pap tests and the data analyses was focused on 566 women that had satisfactory Pap tests with cervical cytology report. The overall mean age was 45 ± 9 years, the youngest participant was 20 years and the oldest was 75 years. Among the study participants, 184 (32.5%) had no formal education or had lead than primary level of completed education, 297 (52.5%) were married or in a co-habiting relationship and 325 (57.4%) had no income or earned less than monthly national minimum wage (<$50USD). A majority 298 (52.7%) of the participants were unaware of the HIV status of their partner, 215 (38.0%) had history of sexually transmitted infections and 57 (10.1%) had used IUCD in the past. Overall, the prevalence of cervical dysplasia was 24% (mild and severe dysplasia were 12.9% and 11.1%, respectively). Based on the Bethesda classification of cervical cytology outcomes, AGC 1(0.2%), ASC-H 4 (0.7%), ASC-US 19 (3.4%), inflammation 20 (3.5%), NILM 410 (72.4%), LSIL 54 (9.5%), and HSIL 58 (10.3%) (Table 1).

**Table 1:** Sociodemographic and clinical characteristics of 566 women with HIV outreach screening project of women with HIV in Jos Nigeria

Baseline Characteristic	*n* (%), *N* = 566
Age Group (Years)	
≤ 34	57 (10.1)
35–45	256 (45.2)
> 45	253 (44.7)
Educational Level	
Primary or less	184 (32.5)
Secondary	229 (40.5)
Post-Secondary	153 (27.0)
Occupation	
Employed	315 (55.7)
Unemployed	251 (44.3)
Monthly Income (USD)	
< 50	325 (57.4)
50–150	208 (36.8)
> 150	33 (5.8)
Marital Status	
Single/Never Married	53 (9.4)
Married/Co-habiting	297 (52.5)
Separate/Divorced/Widowed	216 (38.1)
Housing	
Own	375 (66.3)
Rented	176 (31.1)
Temporary shelter	15 (2.7)
Previous Pregnancy	
Never	31 (5.5)
Yes	535 (94.5)
Multiple Sex Partners	
No	202 (35.7)
Yes	364 (64.3)
Knew HIV Status of Partner	
No	298 (52.7)
Yes	268 (47.3)
History of STI	
No	351 (62.0)
Yes	215 (38.0)
Use of Birth Control Pills	
No	420 (74.2)
Yes	146 (25.8)
Use of IUCD	
No	509 (89.9)
Yes	57 (10.1)
Use of Condom	
No	214 (37.8)
Yes	352 (62.2)
History of Alcohol	
No	420 (74.2)
Yes	146 (25.8)
Smoking	
No	559 (98.8)
Yes	7 (1.2)
Aware of HPV vaccination	
No	523 (92.4)
Yes	43 (7.6)
Co-morbidities	
Diabetes Mellitus	14 (2.5)
Hypertension	84 (14.8)
None	468 (82.7)
History of Pap Test	
First (Under-screened)	462 (81.6)
Second (Screened)	73 (12.9)
≥3 (Screened)	31 (5.5)
Awareness of Pap Test	
No	272 (48.1)
Yes	294 (51.9)
Previous screening (Any type)	
No	455 (80.4)
Yes	111 (19.6)
Cervical Cytology 1	
AGC	1 (0.2)
ASC-H	4 (0.7)
ASC-US	19 (3.4)
Inflammations	20 (3.5)
NILM	410 (72.4)
LSIL	54 (9.5)
HSIL	58 (10.3)
Cervical Cytology 2	
Normal	430 (76.0)
Mild Dysplasia	73 (12.9)
Severe Dysplasia	63 (11.1)

STI: Sexually Transmitted Infection, IUCD: Intrauterine Contraceptive Device, USD: United States Dollar, percent in parenthesis; AGC: Atypical Glandular Cells, ASC-H: Atypical Squamous Cells cannot exclude HSIL, ASC-US: Atypical Squamous Cells of undetermined Significance, NILM: Negative for intraepithelial lesion or malignancy, LSIL: Low-grade intraepithelial lesion, HSIL: High-grade intraepithelial lesion, percent in parenthesis

**Fig. 1:**
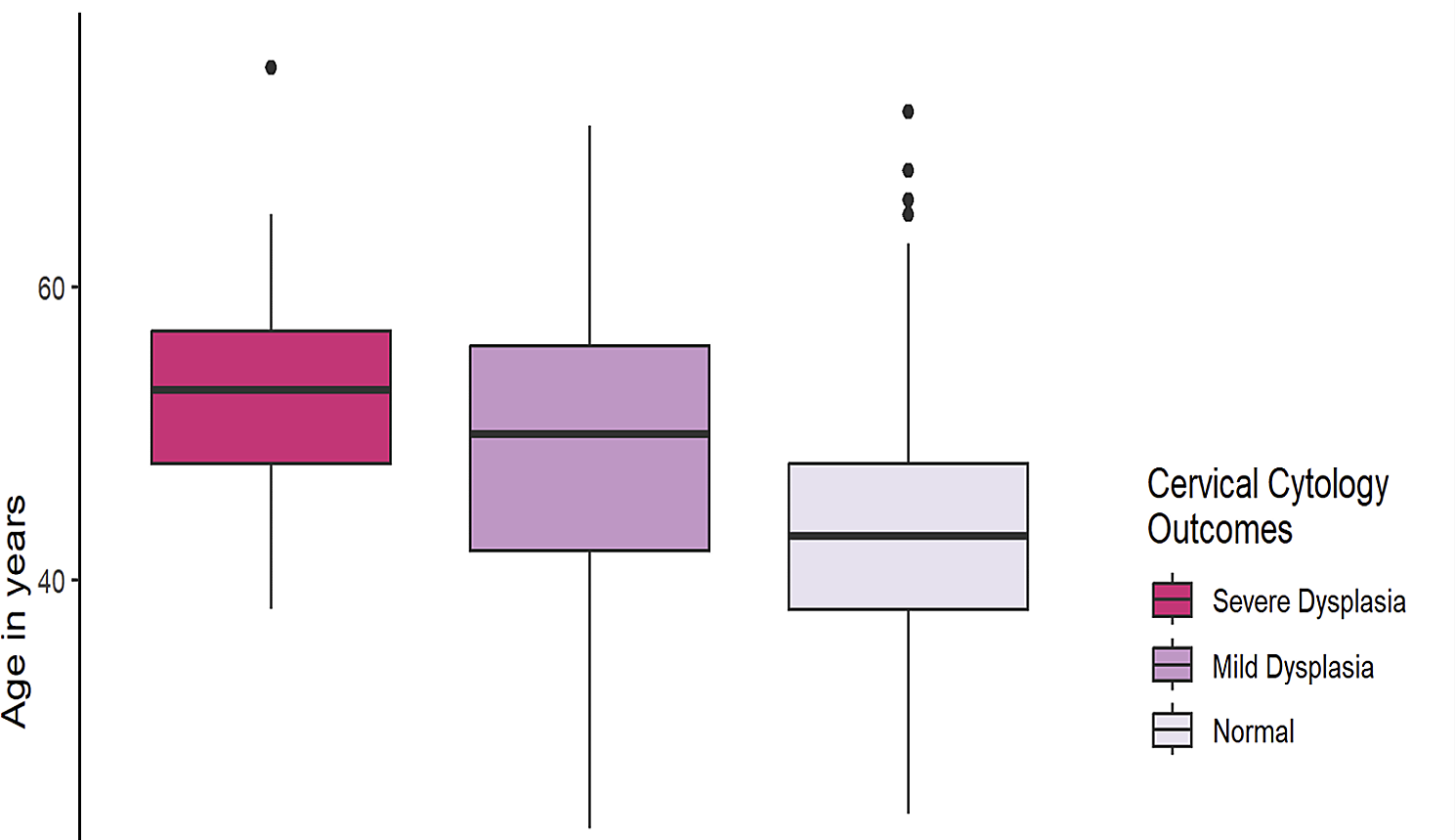
Box Plot of Age at Pap test in women living with HIV in Jos Nigeria

The proportion of study participants who had high-grade cervical dysplasia was significantly higher for women in the age group above 45 years (85.7%) compared with women in the group between 35 and 45 years (14.3%) and those less than 35 years (*p* < 0.000) (Fig. 1).

Women in postmenopausal state (82.5%) had significantly higher proportion of high-grade cervical dysplasia compared with women in premenopausal state (17.5%) (*p* < 0.000), and women who were separated or divorced or widowed 35 (55.6%) had significantly higher proportion of high-grade cervical dysplasia compared to women who were in marital or co-habiting relationship and single or never married women. Similarly, study participants who had not used condom frequently (34 (54.0%) had significantly higher proportion of high-grade precancer compared to those who had used condom, 46 (73.0%) of study participants with no prior history of IUCD use had significantly higher proportion of high-grade precancer compared to those who had history of IUCD usage in the past, and women with no prior Pap test (under-screened) (68.3%, *p* = 0.012) had significantly higher proportion of higher-grade cervical dysplasia compared with women screened in the past (Table 2).

**Table 2:** Baseline socio-demographics versus cervical cytology outcomes in a community outreach project of women with HIV in Jos Nigeria (*n* = 566)

Exposure Indices	Normal (Count, %) (*n* = 430)	Mild Dysplasia (Count, %) (*n* = 73)	Severe Dysplasia (Count, %) (*n* = 63)	*p*-value
Age Group (Years)				
≤34	53 (12.7)	4 (5.5)	0 (0.0)	< 0.000^**b**^
35–45	228 (53.0)	19 (26.0)	9 (14.3)	
>45	149 (34.7)	50 (68.5)	54 (85.7)	
Education Level				
≥ 7 Grade	298 (69.3)	41 (56.2)	42 (66.7)	0.086^**a**^
< 7 Grade	132 (30.7	32 (43.8)	21 (33.3)	
Occupation				
Employed	238 (63.5)	37 (50.7)	40 (63.5)	0.031^**a**^
Unemployed	192 (36.5)	36 (49.3)	23 (36.5)	
Monthly Income (USD)				
< 50	237 (63.5)	48 (65.8)	40 (63.5)	0.33^**b**^
50–150	27 (6.3)	2 (2.7)	4 (6.3)	
> 150	166 (30.2)	23 (31.5)	19 (30.2)	
Marital Status				
Single or Never Married	46 (10.7)	3 (4.1)	4 (6.3)	0.0001^**b**^
Married/Co-habiting	244 (56.7)	29 (39.7)	24 (38.1)	
Separate/Divorced/Widowed	140 (32.6)	41 (56.2)	35 (55.6)	
Menstrual Status				
Post-menopausal	108 (25.1)	47 (64.4)	52 (82.5)	< 0.000^**a**^
Pre-menopausal	322 (74.9)	26 (35.6)	11 (17.5)	
Multiple Sex Partners				
No	154 (35.8)	25 (34.2)	23 (36.5)	0.96^**a**^
Yes	276 (64.2)	48 (65.8)	40 (63.5)	
History of STI				
No	273 (63.5)	42 (57.5)	36 (57.1)	0.44^**a**^
Yes	157 (36.5)	31 (42.5)	27 (42.9)	
Use of Condom				
No	144 (35.5)	36 (49.3)	34 (54.0)	0.0007^**a**^
Yes	286 (66.5)	37 (50.7)	29 (46.0)	
Awareness of Pap Test				
No	208 (48.4)	42 (57.5)	22 (34.9)	0.03^**a**^
Yes	222 (51.6)	31 (42.5)	41 (65.1)	
Use of IUCD				
No	398 (92.6)	65 (89.0)	46 (73.0)	0.001^**a**^
Yes	32 (7.4)	8 (11.0)	17 (27.0)	
Use of Birth Control Pills				
No	238 (76.3)	49 (67.1)	43 (68.3)	0.13^**a**^
Yes	102 (20.7)	24 (32.9)	20 (31.7)	
Knew HIV Status of Partner				
No	213 (49.5)	43 (57.5)	43 (68.3)	0.014^**a**^
Yes	217 (50.5)	20 (42.5)	20 (317)	
History of Pap Test				
Screened	70 (16.3)	14 (19.2)	20 (31.7)	0.012^**a**^
Not-screened	360 (83.7)	59 (80.8)	43 (68.3)	

^***a***^*Pearson’s chi*^*2*^, ^***b***^*Fisher’s Exact, Percent in parenthesis, STI: Sexually Transmitted Infection, IUCD: Intrauterine Contraceptive Device, USD: United States Dollar*

The proportion of study participants who were under-screened (No prior history of Pap test) was 462 (81.6%), with the mean age of 44 years compared to the mean age of 48 years among women who had previous screening (95%CI 1.9, 5.5, *p* = 0.0001). Among the study participants, women above 45 years 214 (46.3%) had significant higher proportion of under-screened status compared to those between the ages of 35–45 years and less than 35 years of age, women with no education or less than primary level of completed education 157 (34.0%) had significantly higher proportion of under-screened status compared to those with secondary or post-secondary completed levels of education. Also, study participants with no income or less than monthly national minimum wage income (<$50.00USD) 250 (54.1%) had significantly higher proportion of under-screened status compared to those that earned more than the monthly minimum national wage. Women who were unaware of Pap test 269 (58.2%) had significantly higher proportion of under-screened status compared to women who were aware of Pap test, women who had no history of sexually transmitted infections 326 (70.6%) had significantly higher proportion of under-screened status compared to women who had history of sexually transmitted infections, and the proportion of WLWH who had no prior Pap test (under-screened) was significantly higher among women with low-grade cervical dysplasia (12.8%) compared to women with high-grade (9.3%) (*p* = 0.013) (Table 3).

**Table 3:** Baseline socio-demographics characteristics by not-screened (under-screened) versus screened in a community outreach project of women with HIV in Jos Nigeria (*n* = 566)

Exposure Indices	Screened (Count, %) (*n* = 104)	Under-screened (Count, %) (*n* = 462)	*p*-value
Age Group (Years)			
≤ 34	4 (3.8)	195 (42.2)	0.011^**b**^
35–45	42 (40.4)	53 (11.5)	
> 45	58 (55.8)	214 (46.3)	
Educational Level			
Primary or less	27 (26.0)	157 (34.0)	0.006^**a**^
Secondary	36 (34.6)	193 (41.8)	
Post-Secondary	41 (39.4)	112 (24.2)	
Occupation			
Unemployed	49(47.1)	202 (43.7)	0.603^**a**^
Employed	55(52.9)	260 (56.3)	
Monthly Income (USD)			
< 50	75 (72.1)	250 (54.1)	0.003^**a**^
50–150	24 (23.1)	184 (39.8)	
> 150	5 (4.8)	28 (6.1)	
Marital Status			
Single/Never Married	4 (3.9)	49 (10.6)	0.099^**b**^
Married/Co-habiting	57 (54.8)	240 (52.0)	
Separate/Divorced/Widowed	43 (41.3)	173 (37.4)	
Multiple Sex Partners			
No	25 (24.0)	177 (38.3)	0.008^**a**^
Yes	79 (76.0)	285 (61.7)	
Awareness of Pap Test			
Yes	101 (97.1)	193 (41.8)	< 0.000^**b**^
No	3 (2.9)	269 (58.2)	
History of STI			
No	25 (24.0)	326 (70.6)	< 0.000^**a**^
Yes	79 (76.0)	136 (29.4)	
Use Birth Control Pills			
No	66 (63.5)	354 (76.6)	0.008^**a**^
Yes	38 (36.5)	108 (23.4)	
History of Alcohol			
No	68 (65.4)	352 (76.2)	0.031^**a**^
Yes	36 (34.6)	110 (23.8)	
Knew HIV Status of Partner			
No	85 (33.85)	213 (46.1)	< 0.000^**a**^
Yes	19 (66.15)	249 (53.9)	
Use of IUCD			
No	83 (79.8)	426 (92.2)	0.0003^**a**^
Yes	21 (20.2)	36 (7.8)	
Use of Condom			
Yes	80 (76.9)	272 (58.9)	0.0009^**a**^
No	24 (23.1)	190 (41.1)	
Co-morbidities			
Diabetes Mellitus	4 (3.8)	10 (2.2)	0.0001^**b**^
Hypertension	31 (29.8)	53 (1.5)	
None	69 (66.4)	399 (86.3)	
Cervical Cytology			
Normal	70 (67.3)	360 (77.9)	0.013^**a**^
Mild Dysplasia	14 (13.5)	59 (12.8)	
Severe Dysplasia	20 (19.2)	43 (9.3)	

^***a***^*Pearson’s chi*^*2*^, ^***b***^*Fisher’s Exact, Percent in parenthesis, STI: Sexually Transmitted Infection, IUCD: Intrauterine Contraceptive Device, USD: United States Dollar*

### Unadjusted and adjusted logistic regression model of independent variables with mild cervical dysplasia

For the crude logistic regression, monthly income was not significantly associated with first Pap test (OR = 1.56, 95%CI 0.94, 2.66). Age of women above 45 years (OR = 4.10, 95%CI 2.43–7.09), level of education completed below grade 7 (OR = 1.78, 95%CI 1.07–2.95), women who were separated, divorced or widowed (OR = 4.49, 95%CI 1.54–19.51), being postmenopausal (OR = 5.39, 95%CI 3.21–9.23), and women with no history of condom use (OR = 1.93, 95%CI 1.17–3.19) were significantly associated with developing mild cervical dysplasia (Table 4).

**Table 4:** Bivariate and multivariate Logistic regression model with unadjusted and adjusted odds ratio of the association between age above 45 years, other socio-demographic factors with the likelihood of mild cervical dysplasia in a community outreach project of women with HIV in Jos Nigeria (*n* = 566)

Variable	OR (95%CI)	p-value	aOR (95%CI)	*p*-value
Age (Years)				
≤ 45	1			
> 45	4.10 (2.43, 7.09)	0.0001^*^	1.61 (0.78, 3.33)	0.19
Education Status				
≥7	1			
<7	1.78 (1.07, 2.95)	0.025^*^	-	-
Occupation				
Employed	1			
Unemployed	1.21 (0.73, 1.98)	0.46	-	-
Monthly Income (USD)				
≥50	1			
<50	1.56 (0.94, 2.66)	0.09	-	-
Marital Status				
Single	1			
Married	1.82 (0.61, 7.80)	0.34		
Separated/Divorced/Widow	4.49 (1.54, 19.15)	0.016^*^	-	-
Monogamous Co-habituating				
Yes	1			
No	1.71 (1.04, 2.85)	0.036^*^	-	-
Menstrual Status				
Pre-menopausal	1			
Post-menopausal	5.39 (3.21, 9.23)	0.000^*^	4.39 (2.17, 9.24)	0.0001^*^
History of smoking				
No	1			
Yes	2.39 (0.34, 11.34)	0.302	5.39 (0.73, 27.19)	0.054
Knew HIV Status of Partner				
Yes	1			
No	1.38 (0.84, 2.29)	0.207	1.62 (0.95, 2.79)	0.076
History of STI				
No	1			
Yes	1.28 (0.77, 2.12)	0.33	-	-
Use of IUCD				
Yes	1			
No	1.53 (0.63, 3.32)	0.31	-	-
Use of Condom				
Yes	1			
No	1.93 (1.17, 3.19)	0.01^*^	-	-
Use of Birth Control Pills				
No	1			
Yes	1.58 (0.91, 2.67)	0.097	-	-
Co-morbidities				
No	1			
Yes	1.84 (0.99, 3.28)	0.045^*^	-	-
History of Pap Test				
Screened	1			
Not-screened	1.42 (0.90, 2.14)	0.11	-	-

STI: Sexually Transmitted Infection, IUCD: Intrauterine Contraceptive Device, *Significant association, USD: United States Dollar

In the adjusted logistic regression, being in a postmenopausal state (OR = 4.39, 95%CI 2.17–9.24), was independently associated with developing mild dysplasia.

### Unadjusted and adjusted logistic regression model of independent variables with severe cervical dysplasia

The unadjusted logistic regression, educational completion of < grade 7 was not significantly associated with severe dysplasia (OR = 1.14, 95%CI 0.64, 1.98). Age of women above 45 years (OR = 11.32, 95%CI 5.70–25.12), postmenopausal state (OR = 14.09, 95%CI 7.36–29.37), women with no history of IUCD use (OR = 4.60, 95%CI 2.33–8.85), women with no history of condom use (OR = 2.33, 95%CI 1.37–3.99), and women with no prior Pap test (OR = 1.33, 95%CI 1.33–3.04), were significantly associated with severe cervical dysplasia. However, being aware of cancer screening (OR = 0.57, 95%CI 0.33–0.99), was associated with less likelihood of developing severe dysplasia (Table 5).

**Table 5:** Bivariate and multivariate Logistic regression model with unadjusted and adjusted odds ratio of the association between age above 45 years, other socio-demographic factors with the likelihood of severe cervical dysplasia in a community outreach project of women with HIV in Jos Nigeria (*n* = 566)

Variable	OR (95%CI)	*p*-value	aOR (95%CI)	*p*-value
Age (Years)				
≤ 45	1			
> 45	11.32 (5.70, 25.12)	0.0001^*^	3.48(1.39, 9.21)	0.009
Education Status				
≥7	1			
<7	1.14 (0.64, 1.98)	0.64	-	-
Occupation				
Employed	1			
Unemployed	0.71 (0.41, 1.22)	0.22	0.44 (0.22, 0.85)	0.016^*^
Monthly Income (USD)				
≥50	1			
<50	1.42 (0.83, 2.48)	0.21	-	-
Marital Status				
Single	1			
Married	1.13 (0.41, 3.98)	0.83		
Separated/Divorced/Widow	2.88 (1.08, 9.99)	0.06	-	-
Monogamous Co-habituating				
Yes	1			
No	1.59 (0.94, 2.74)	0.09	-	
Menstrual Status				
Pre-menopausal	1			
Post-menopausal	14.9 (7.36, 29.37)	0.000^*^	7.69 (3.37, 19.15)	0.000^*^
Use of Alcohol				
No	1			
Yes	1.21 (0.66, 2.14)	0.53	-	-
Aware of CCS				
No	1			
Yes	0.57 (0.33, 0.99)	0.048^*^	-	-
Knew HIV Status of Partner				
Yes	1			
No	2.19 (1.26, 3.92)	0.006^*^	2.52 (1.33, 4.89)	0.005^*^
History of STI				
No	1,0			
Yes	1.30 (0.76, 2.22)	0.33	-	-
Use of IUCD				
Yes	1			
No	4.60 (2.33, 8.85)	0.0001^*^	5.94 (2.49, 14.42)	0.0001^*^
Use of Condom				
Yes	1			
No	2.33 (1.37, 3.99)	0.002^*^	-	-
Use of Birth Control Pills				
No	1			
Yes	1.50 (0.83, 2.63)	0.17	-	-
Co-morbidities				
No	1			
Yes	1.75 (0.90, 3.25)	0.08	-	-
History of Pap Test				
Screened	1			
Not-screened	2.03 (1.33, 3.04)	0.0007^*^	-	-

STI: Sexually Transmitted Infection, IUCD: Intrauterine Contraceptive Device, *Significant association, USD: United States Dollar

**Fig. 2:**
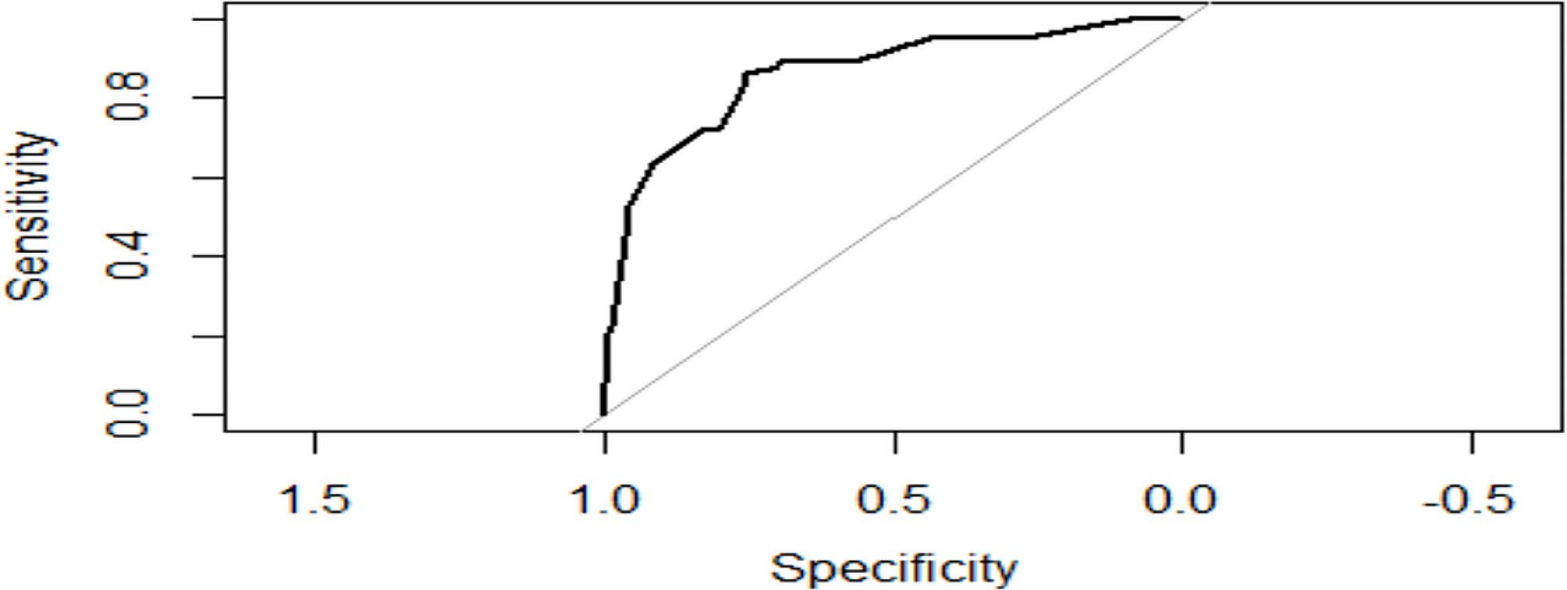
Receiver Operator Characteristic (ROC) Curves for the Predictive Model of Women who had Severe Dysplasia Area Under the curve (AUC): 0.86

In the adjusted logistic regression, age of women above 45 years (OR = 3.48, 95%CI 1.39–9.21), being in postmenopausal state (OR = 7.69, 95%CI 3.37–19.15), knowing HIV status of sex partner (OR = 2.52, 95%CI 1.33–4.89), and women with no history of IUCD use (OR = 5.94, 95%CI 2.49–14.42) were independently associated with developing severe dysplasia with a strong predictive power of 0.86 (Fig. 2).

### Unadjusted and adjusted logistic regression model of independent variables with prior history of Pap test (screened) women

In the crude logistic regression, age 35–45 years was not significantly associated with first Pap test (OR = 0.38, 95%CI 0.11, 1.00). Age of women above 45 years (OR = 0.25, 95%CI 0.07–0.65), with monthly income > 50 USD (OR = 0.46, 95%CI 0.28–0.72), prior history of STI (OR = 0.13, 95%CI 0.08–0.21), prior use of IUCD (OR = 0.33, 95%CI 0.19–0.61), and severe cervical dysplasia (OR = 0.42, 95%CI 0.23–0.77), were more likely to have had their Pap test in the past (Table 6).

**Table 6:** Bivariate and multivariate Logistic regression model with unadjusted and adjusted odds ratio of the association between cervical cytology outcomes, other socio-demographic factors with the likelihood of being screened in a community outreach project of women with HIV in Jos Nigeria (*n* = 566)

Variable	OR (95%CI)	p-value	aOR (95%CI)	p-value
Age (Years)				
≤ 34	1			
35–45	0.38 (0.11,1.00)	0.079		
> 45	0.25 (0.07, 0.65)	0.011^*^	-	-
Education Status				
≥7	1			
<7	1.47 (0.92, 2.40)	0.116	-	-
Occupation				
Employed	1			
Unemployed	0.87 (0.57, 1.34)	0.53	-	-
Monthly Income (USD)				
<50	1			
≥50	0.46 (0.28, 0.72)	0.001^*^	0.91(0.51, 1.61)	0.74
Marital Status				
Single	1			
Married	0.34 (0.10, 0.89)	0.048^*^		
Separated/Divorced/Widow	0.33 (0.09, 0.86)	0.042^*^	-	-
Menstrual Status				
Pre-menopausal	1			
Post-menopausal	0.64 (0.42, 0.99)	0.044^*^	0.54(0.29, 1.01)	0.054
History of Pregnancy				
Never	1			
Yes	0.29 (0.05, 0.99)	0.09	0.24(0.03, 0.98)	0.08
Use of Alcohol				
No	1			
Yes	0.59 (0.38, 0.94)	0.024^*^	-	-
Multiple Sex Partners				
No	1			
Yes	0.51 (0.31, 0.82)	0.007^*^	-	-
History of STI				
No	1			
Yes	0.13 (0.08, 0.21)	0.000^*^	0.17(0.10, 0.29)	0.000^*^
Use of IUCD				
No	1			
Yes	0.33 (0.19, 0.61)	0.0002^*^	0.32(0.15, 0.71)	0.004^*^
Use of Condom				
No	1			
Yes	2.33 (1.44, 3.88)	0.0008^*^	2.50(1.40, 4.62)	0.003^*^
Knew HIV Status Partner				
No	1			
Yes	0.19 (0.11, 0.32)	0.000^*^	0.27(0.15, 0.49)	0.0002^*^
Co-morbidities				
No	1			
Yes	0.31 (0.19, 0.51)	0.0001^*^	0.46(0.26, 0.83)	0.009^*^
Cervical Cytology Report				
Normal	1			
Mild Dysplasia	0.82 (0.44, 1.60)	0.539	1.50(0.67, 3.52)	0.33
Severe Dysplasia	0.42 (0.23, 0.77)	0.004^*^	0.88(0.38, 2.13)	0.77

STI: Sexually Transmitted Infection, IUCD: Intrauterine Contraceptive Device, *Significant association, USD: United States Dollar

**Fig. 3:**
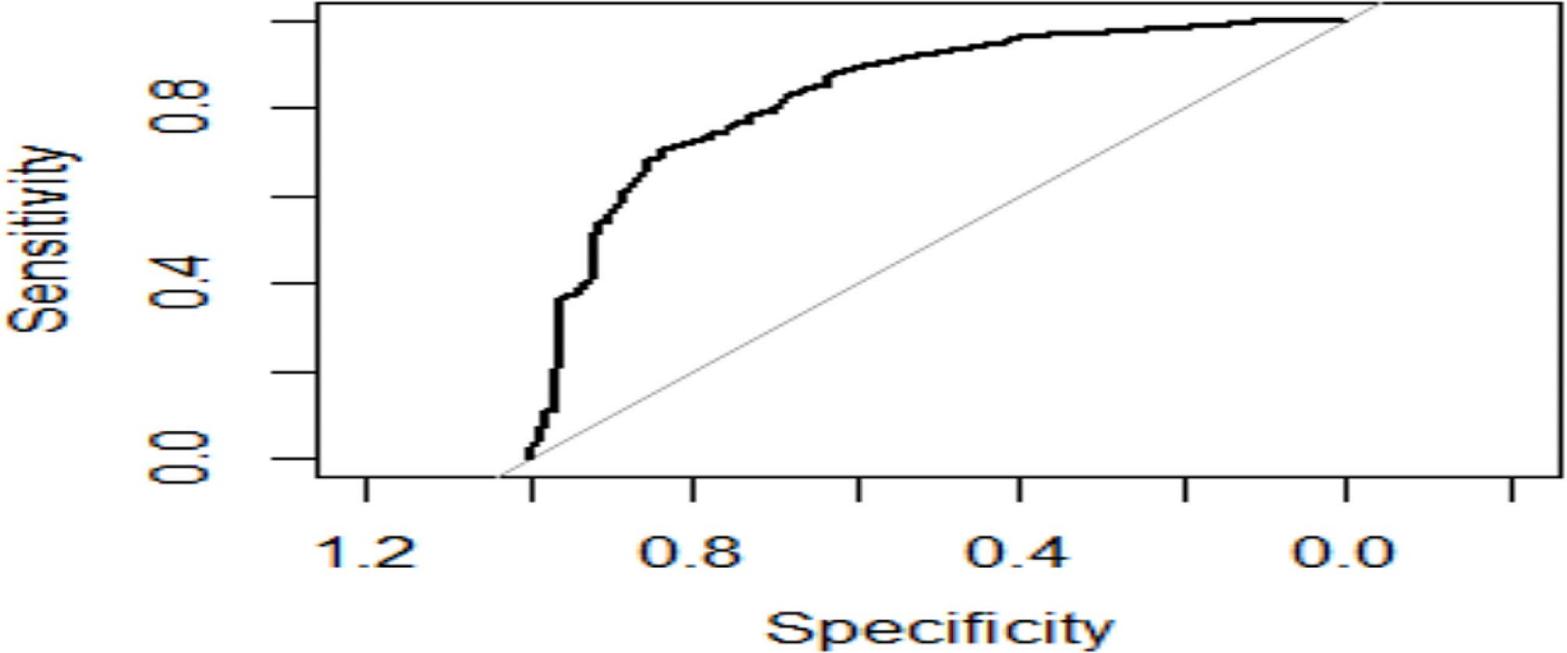
Receiver Operator Characteristic (ROC) Curves for the Predictive Model of Women with prior Pap test Area Under the Curve (AUC): 0.84

For the adjusted logistic regression, history of STI (OR = 0.17, 95%CI 0.10–0.29), prior use of IUCD (OR = 0.32, 95%CI 0.15–0.71), prior use of condom (OR = 2.50, 95%CI 1.39–4.62), knowing HIV status of partner (OR = 0.27, 95%CI 0.15–0.49) and history of co-morbidities (OR = 0.46, 95%CI 0.26–0.83) were independently associated with likelihood of women who had Pap test in the past with a strong predictive power of 0.84 (Fig. 3).

## Discussion

The findings in this study showed that the prevalence of cervical dysplasia was high, specifically, the prevalence of severe dysplasia which was significantly different among unemployed women above 45 years and post-menopausal, who are separated or widowed or divorced and screening for the first time. The same findings were found in the study among HIV-infected women in an urban, United States safety-net healthcare system Parkland hospital in Dallas, USA, and urban community in Delhi, India [4, 17]. These findings are important, considering the high risk for CC among WLWH, the intervention provided to prevent progression to CC in the future, and the need to provide continuous screening to reduce CC incidence and mortality in low-resource settings.

Postmenopausal women with HIV who were separated or widowed or divorced and above 45 years of age with less education had more likelihood for developing mild dysplasia, and postmenopausal status was independently associated with mild cervical dysplasia. Although, mild dysplasia is a low grade pre-cancerous lesion, the immune compromise status of WLWH puts them at higher risk for progression to severe dysplasia and invasive cervical cancer within a shorter duration [18]. The same findings were noted in a previous study, and the common denominator was the immune compromise status with HIV infection, other factors responsible for the progression to higher grade were unknown [11], hence the importance of regular screening among the high-risk population of WLWH.

Similarly, postmenopausal women above 45 years, who were unaware of cervical cancer screening, with prior use of IUCD and use of condom had lower odds for developing severe cervical dysplasia. The same findings, especially the use of IUCD and age at screening were associated with developing cervical dysplasia [7, 19]. The goal for the screening is to identify severe dysplasia, equivalent to high grade precancerous lesion that requires immediate intervention or treatment as the chances of reversal are very unlikely. Unlike HIV negative women, the progression from severe dysplasia to cervical cancer is faster among WLWH and likely to present in advance stages of the cervical cancer [18]. Identifying women with severe dysplasia is important as this will reduce CC incidence by preventing progression to CC among WLWH in Jos, Nigeria.

The proportion of under-screened women despite being on antiretroviral therapy in the HIV clinic for a long period was high. Women living with HIV who were younger below 45 years, low socio-economic status, without prior history of IUCD usage and without history for STI were more likely to be under-screened. The same findings were shown in United States safety-net healthcare system Parkland hospital in Dallas, USA [4]. Although, the study in United States safety-net healthcare system was longitudinal, population studied shared similar characteristics with our study as women who were living with HIV and of low socio-economic status, knowing that CC is a disease of those with low socio-economic status [20].

Similarly, the same findings were reported in a study in India, which showed high prevalence of high-grade lesions among WLWH. Cervical cancer is considered an AIDS-defining illness hence, regular screening was suggested for early detection of precancerous and invasive disease. This is even more important in the era of ART, which can increase life expectancy of WLWH but does not prevent persistence of HPV infection with increased risk of CC and associated mortality even if they had survived the scourge of other conditions associated with AIDS [17].

This study has provided data on burden of cervical dysplasia, predictors of mild and severe cervical dysplasia, and barriers to accessing CCS among WLWH in Jos, Nigeria. The findings in this project will be useful for stakeholders in HIV/AIDS care programs to advocate for policies on change management in workings around HIV clinics for the inclusion of CCS services. Streamlining CCS into HIV care services may increase CCS coverage with resultant identification of cervical dysplasia, early CC, and treatment of pre-cancer and early CC to reduce CC incidence and mortality.

However, the study is limited by the bias in population selection using indigent women in HIV clinic used as study site. Also, missing variables on HIV diagnosis, stage of HIV/AIDS, and treatment, age at sexual debut, parity, and age at first childbirth. In addition, a longitudinal study design would have provided better evidence on effect of HIV on CCS outcomes.

## Conclusion

The prevalence of cervical dysplasia and under-screened women were high among WLWH in Jos, Nigeria. Lack of organized screening and under-screening among indigent women living with HIV who are at a higher risk for pre-cervical cancer accounts for the persistent increased in incidence of CC and reversal of gains made in HIV/AIDS control and prevention in low-resource settings.

Therefore, ending the intersecting epidemics of HIV/AIDS and cervical cancer by 2030 global target in low-resource settings might not be feasible, unless coverage for CCS among women with HIV is made more accessible. Women with HIV should have access to services for CCS at HIV diagnosis or ART initiation in HIV endemic and low-resource settings.

## Data Availability

The dataset from this study was collected and stored in the electronic institutional REDCap database of the University of Jos and is available for sharing through the corresponding author on request.

## Abbreviations

AGC: Atypical glandular cells
AIC: Akaike Information Criterion
AIDS: Acquired Immunodeficiency Syndrome
ART: Antiretroviral therapy
ASC-H: Atypical squamous cell cannot exclude HSIL
ASC-US: Atypical squamous cell of undetermined significance
BHUTH: Bingham University Teaching Hospital
BIC: Bayesian Information Criterion
BMI: Body Mass Index
CC: Cervical Cancer
CCS: Cervical Cancer Screening
HIV: Human Immunodeficiency Virus
HPV: Human Papillomavirus
HSIL: High-grade squamous intraepithelial lesion
IDSA: Infectious Disease Society of America
IRB: Institutional Review Board
IUCD: Intrauterine contraceptive device
JUTH: Jos University Teaching Hospital
LSIL: Low-grade squamous intraepithelial lesion
NILM: Negative for intraepithelial lesion or malignancy
OLA: Our Lady of Apostle
Pap: Papanicolaou
PLHIV: People living with HIV
PSSH: Plateau State Specialist Hospital
SIL: Squamous intraepithelial lesion
SSA: Sub-Saharan Africa
STI: Sexually Transmitted Infection
USA: United States of America
USD: United States Dollar
WLWH: Women living with HIV
